# Genetic Variability of HIV-1 for Drug Resistance Assay Development

**DOI:** 10.3390/v8020048

**Published:** 2016-02-11

**Authors:** Dana S. Clutter, Patricia Rojas Sánchez, Soo-Yon Rhee, Robert W. Shafer

**Affiliations:** 1 Division of Infectious Diseases and Geographic Medicine, Stanford University School of Medicine, 300 Pasteur Drive, L-134, Stanford, CA 94035, USA; syrhee@stanford.edu (S.-Y.R.); rshafer@stanford.edu (R.W.S.); 2 HIV-1 Molecular Epidemiology Laboratory, Microbiology and Parasitology Department, Hospital Ramón y Cajal-IRYCIS and CIBER-ESP, Madrid 28034, Spain; patriciarrojas@gmail.com

**Keywords:** HIV-1, drug resistance mutation, variability, point-of-care

## Abstract

A hybridization-based point-of-care (POC) assay for HIV-1 drug resistance would be useful in low- and middle-income countries (LMICs) where resistance testing is not routinely available. The major obstacle in developing such an assay is the extreme genetic variability of HIV-1. We analyzed 27,203 reverse transcriptase (RT) sequences from the Stanford HIV Drug Resistance Database originating from six LMIC regions. We characterized the variability in a 27-nucleotide window surrounding six clinically important drug resistance mutations (DRMs) at positions 65, 103, 106, 181, 184, and 190. The number of distinct codons at each DRM position ranged from four at position 184 to 11 at position 190. Depending on the mutation, between 11 and 15 of the 24 flanking nucleotide positions were variable. Nonetheless, most flanking sequences differed from a core set of 10 flanking sequences by just one or two nucleotides. Flanking sequence variability was also lower in each LMIC region compared with overall variability in all regions. We also describe an online program that we developed to perform similar analyses for mutations at any position in RT, protease, or integrase.

## 1. Introduction

The increasing prevalence of acquired and transmitted HIV-1 drug resistance is an obstacle to successful antiretroviral (ARV) therapy in the low- and middle-income countries (LMICs) hardest hit by the HIV-1 pandemic [1]. Genotypic drug resistance testing could facilitate the choice of initial ARV therapy in areas with rising transmitted drug resistance (TDR) and enable care-providers to determine which individuals with virological failure on a first- or second-line ARV regimen require a treatment change. Despite the decreasing costs of standard genotypic resistance testing and next-generation sequencing (NGS), these assays remain prohibitively complex and costly for many LMICs [2,3]. Additionally, the dependency on batching samples to reduce the cost of NGS is a disadvantage when timeliness is desired [4]. An inexpensive point-of-care (POC) genotypic resistance test would be useful in settings where the resources, capacity, and infrastructure to perform standard genotypic drug resistance testing or NGS are limited. A POC genotypic resistance test would be particularly useful in conjunction with the POC HIV-1 viral load tests that are currently being introduced in LMICs [5,6,7].

A POC genotypic resistance test is likely to involve the use of a hybridization-based point mutation assay for detecting the most clinically significant drug-resistance mutations (DRMs) [8,9,10,11]. Preliminary data suggests that a set of six reverse transcriptase (RT) DRMs—the nucleoside reverse transcriptase inhibitor (NRTI)-associated DRMs K65R and M184V and the non-nucleoside reverse transcriptase inhibitor (NNRTI)-associated DRMs K103N, V106M, Y181C and G190A—are about 60% sensitive for detecting intermediate or high-level TDR and 99% sensitive for detecting intermediate or high-level acquired drug resistance (ADR) in patients with virological failure on a first-line WHO recommended NRTI/NNRTI containing regimen [12]. The major obstacle to the development of a hybridization-based assay is the extreme genetic variability of HIV-1 [11,13]. Here we characterize the genetic variability at and surrounding each of the six DRMs mentioned above and introduce a web-based program that allows researchers to perform analyses similar to those we present here.

## 2. Materials and Methods

### 2.1. Sequence Selection

We analyzed group M HIV-1 plasma RT sequences from the Stanford HIV Drug Resistance Database (HIVDB) [14]. Sequences were characterized by the country of origin and year of collection. Sequences were assigned to one of the following six LMIC regions: Southern Africa, Central Africa, Eastern Africa, Western Africa, India, and the LMICs of South and Southeast Asia [15]. Isolates were assigned a subtype using the Rega Subtyping tool and the annotation provided by authors.

### 2.2. Analysis of Codons

Codon variability was characterized by the proportions of distinct nucleotide triplets encoding either wild type or mutant residues at each DRM position. Because there are well known examples of inter-subtype differences in the proportions of codons at several drug-resistance positions [16], we examined codon variability within each of the seven most common subtypes: A, B, C, D, G, CRF01_AE, and CRF02_AG. Codons that included electrophoretic nucleotide mixtures were not included.

### 2.3. Analysis of Flanking Segments

We examined a span of 27 nucleotides encompassing each drug-resistance position as well as 12 upstream and 12 downstream nucleotides. These flanking nucleotides are important for hybridization strategies relying on a terminal 3’ mismatch for either positive or negative-stranded cDNA and for those that rely on a central mismatch [11,17,18].

We defined the positional variability of flanking segments—the 12 upstream and downstream nucleotides—as the proportions of nucleotides at each of the 24 flanking nucleic acid positions. To represent positional variability, we generated sequence logos with heights proportional to the information content at each nucleotide position [19].

We defined the segmental variability of flanking segments as the distribution of distinct haplotypes flanking each DRM position. For this analysis, we determined how many distinct haplotypes were present in the complete dataset and the extent to which haplotype diversity segregated with geographic region. The 10, 25, and 100 most common haplotypes were referred to as universal if they were from the complete set of sequences from the six LMIC regions or regional if there were from one of the six LMIC regions.

## 3. Results

### 3.1. Sequences

**Figure 1:**
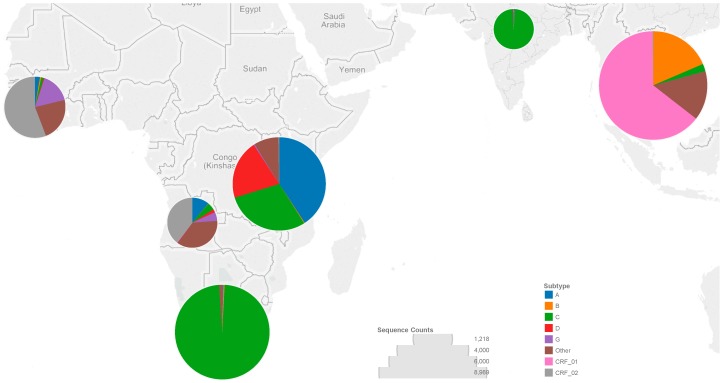
The number of sequences from each low- and middle-income country (LMIC) region corresponds to the diameter of the circle overlying each region, as indicated by the circle diameters in the “Sequence Counts” legend. The colors making up each circle correspond to the proportion of each subtype or circulating recombinant form (CRF) in that region, as indicated in the “Subtype” legend.

We analyzed 27,203 HIV-1 RT sequences from as many individuals from six LMIC regions. Overall, 32% of sequences were from the LMICs of South and Southeast Asia, 22% from Southern Africa, 25% from Eastern Africa, 10% from Western Africa, 7% from Central Africa, and 4% from India (Figure 1). The most common subtypes were subtype C (35%), CRF01_AE (21%), A (11%), CRF02_AG (9%), B (6%), D (5%), and G (2%). Less common subtypes or circulating recombinant forms (CRFs) comprised 11% of sequences. Sequences were from 18,564 (68%) untreated individuals, 7551 (28%) treated individuals and 1088 (4%) individuals with unknown treatment status.

### 3.2. Codons

Table 1 shows the proportions of distinct wild type and mutant codons at each DRM position present in ≥1% of sequences for any of the seven most common subtypes or CRFs. In addition to K65R, K103N, V106M, Y181C, M184V, and G190A, these six positions also encode the following less common DRMs: K65N, K103S, V106A, Y181I/V, M184I, and G190S/E/Q and two polymorphic mutations, K103R and V106I, that do not confer significant drug resistance. The total number of distinct wild type and mutant codons at each DRM position ranged from four for position 184 to 11 for position 190.

At position 65, the wild type lysine (K) is encoded by AAG in 99% of subtype C sequences but by AAA in >95% of the sequences of the other subtypes. At position 106, the wild type valine (V) is encoded by GTG in 87% of subtype C sequences but by GTA in >85% of other subtype sequences. At position 181, the wild type tyrosine (Y) is encoded by TAC in >90% of subtypes G and CRF02_AG sequences but by TAT in >95% of other subtype sequences. Each of these silent nucleotide changes results in a predisposition for a different subtype-specific mutant variant. At position 106, this predisposition leads to an increased prevalence of the DRM V106M in subtype C viruses (Table 1; [20]). In most other subtypes the dominant mutation is V106A, which results in intermediate efavirenz and high-level nevirapine resistance, whereas V106M results in high-level resistance to both NNRTIs [14,21].

**Table 1:** Wild type and mutant codon frequency by subtype.

DRM Position	Codon	AA	A *n* = 2968	B *n* = 1725	C *n* = 9405	D *n* = 1355	G *n* = 597	CRF_01 *n* = 5590	CRF_02 *n* = 2342
**65**	*WT (23,365; 98.1)*								
	AAA	K	96.7	97.8	0.9	97.6	98.8	98.9	97.7
	AAG	K	3.3	2.2	**99.1**	2.4	1.2	1.1	2.4
	*Mutant (446; 1.9)*								
	AGA	R	84.6	100	3.7	100	100	85.9	93.1
	AGG	R	7.7	0	**95.6**	0	0	1.9	3.5
	AAT	N	7.7	0	0.7	0	0	2.8	0
	AAC	N	0	0	0	0	0	9.4	3.5
	*Total coverage*		100.0	100.0	100.0	100.0	100.0	100.0	100.0
**103**	*WT (20,748; 89.8)*								
	AAA	K	95.6	95.7	91.6	96.5	92.2	95.7	98.0
	AAG	K	4.2	2.1	6.4	3.2	6.8	3.8	1.7
	AGA	R	0.3	2.3	2.0	0.2	1.0	0.6	0.2
	*Mutant (2369; 10.3)*								
	AAC	N	84.1	77.8	77.3	75.5	80.8	77.6	82.6
	AAT	N	11.2	17.8	18.5	20.4	19.2	19.2	16.9
	AGC	S	4.7	4.3	4.3	2.0	0	2.5	0.5
	ACA	T	0	0	0	2.0	0	0.7	0
	*Total coverage*		99.83	99.58	99.65	99.92	99.28	99.64	99.96
**106**	*WT (22,427; 96.0)*								
	GTA	V	97.51	90.1	13.3	95.4	96.2	86.4	97.4
	GTG	V	1.7	2.6	**86.6**	4.0	1.2	8.5	1.9
	ATA	I	0.8	7.4	0.2	0.6	2.6	5.2	0.7
	*Mutant (926; 3.4)*								
	GCA	A	85.7	70.8	0.4	80.0	90.9	37.5	75.0
	GCG	A	0	0	2.4	0	0	0	0
	ATG	M	14.3	29.2	**97.2**	20.0	9.1	62.5	25.0
	*Total coverage*		99.49	99.64	99.34	99.55	100	99.48	99.66
**181**	*WT (21,972; 93.5)*								
	TAT	Y	95.7	97.5	96.3	95.5	10.0	98.4	8.6
	TAC	Y	4.3	2.5	3.8	4.5	**90.0**	1.6	**91.4**
	*Mutant (1541; 6.6)*								
	TGT	C	81.8	96.4	88.3	88.4	9.4	86.5	8.5
	TGC	C	7.3	0.9	4.3	4.7	**87.1**	3.0	**87.3**
	ATT	I	5.5	0.9	3.9	0	0	4.8	0
	ATC	I	0	0.9	0.2	0	2.4	0.2	2.1
	GTT	V	5.5	0.9	3.2	7.0	0	5.5	0.7
	GTC	V	0	0	0	0	1.2	0.2	1.4
	*Total coverage*		100.0	99.9	100.0	100.0	99.8	100.0	100.0
**184**	*WT (19,231; 81.0)*								
	ATG	M	100.0	100.0	100.0	100.0	100.0	100.0	100.0
	*Mutant (4498; 19.0)*								
	GTG	V	90.3	78.1	90.2	89.4	83.3	81.5	87.6
	GTA	V	9.7	7.3	6.9	9.6	15.4	14.7	10.4
	ATA	I	0	14.6	3.0	1.0	1.3	3.8	2.0
	*Total coverage*		100.0	99.5	99.9	99.9	99.8	99.8	99.9
**190**	*WT (22,097; 94.7)*								
	GGA	G	95.2	95.3	95.4	96.4	90.1	94.6	92.8
	GGC	G	1.5	3.2	1.3	0.5	1.3	3.4	1.6
	GGG	G	3.3	1.5	3.3	3.1	8.6	2.0	5.7
	*Mutant (1243; 5.3)*								
	GCA	A	92.9	70.7	83.4	89.7	92.3	87.9	89.0
	GCG	A	0	1.2	1.7	3.5	2.6	2.2	1.4
	GCC	A	0	2.4	0.8	0	0	1.2	0
	AGC	S	2.9	24.4	3.9	3.5	2.6	2.7	2.7
	AGT	S	0	1.2	1.9	0	2.6	1.9	2.7
	TCA	S	1.4	0	0.9	0	0	1.5	1.4
	GAA	E	2.9	0	4.5	0	0	1.7	2.7
	CAA	Q	0	0	3.0	3.5	0	1.0	0
	*Total coverage*		99.7	99.9	99.5	99.6	99.1	99.8	99.8

The frequency of all codons of at least 1% frequency in any of the seven most common subtypes or circulating recombinant forms (CRFs) are shown by subtype for both wild type and mutant codons. The 23,982 sequences from the most seven most common subtypes or CRFs were included in this analysis. Within the analysis of each drug resistance mutation (DRM position, sequences bearing mixtures in the codon of interest were excluded. The number and proportion of wild type and mutant sequences used in the analysis of each DRM are listed in the Codon columns (N; %). Total coverage represents the number of codons from all sequences in the database of that subtype that would match one of the codons listed here for that DRM position. Notable inter-subtype differences in codon frequencies appear in bold font. Abbreviations: AA, amino acid; WT, wild type.

### 3.3. Flanking Segments

**Figure 2:**
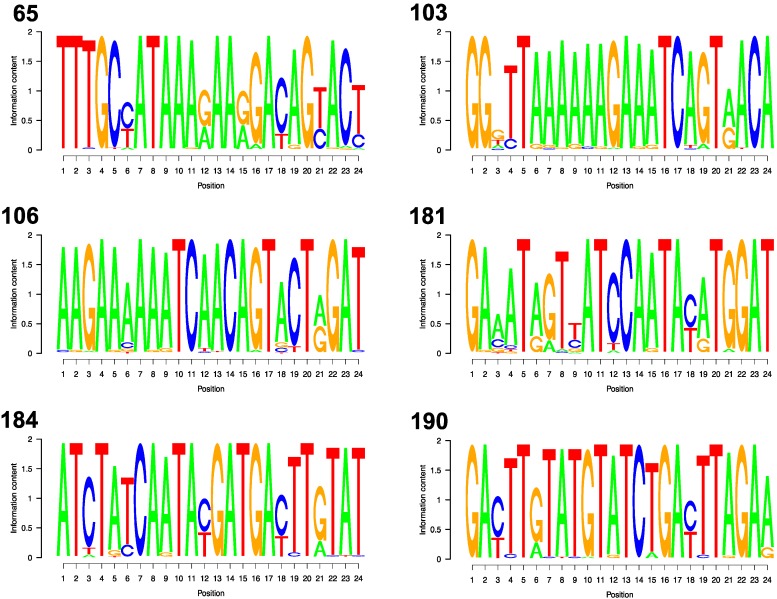
For each DRM Position, the variability at each of the 24 nucleotide positions making up the flanking segments are shown. The letter(s) shown at each nucleotide position indicate which of the four nucleotides are found at that position among all low and middle income country sequences. The relative sizes of the letters indicate their frequency at the position that was weighted by the information content of the position. Therefore, the combined height of the letter(s) in each nucleotide position corresponds to the information content of the position.

Figure 2 shows the sequence logos for each of the six DRM codons. The mean information content per position was 1.72 surrounding position 65, 1.77 for position 103, 1.82 for position 106, 1.68 for 181, 1.79 for 184, and 1.81 for 190. Depending on the DRM, between 11 and 15 of the 24 flanking nucleotide positions were variable, defined as having two or more nucleotides with ≥1% prevalence: codon 65 (13 variable positions), codon 103 (15 variable positions), codon 106 (14 variable positions), codon 181 (12 variable positions), codon 184 (11 variable positions), and codon 190 (13 variable positions). However, at most variable nucleotide positions (58% to 85% depending on the DRM) variability resulted only from transitions (the presence of A and G or C and T) which would result in mismatched base pairs (A:C and G:T) that do not cause the most severe disruption of hybridization [22,23].

**Figure 3:**
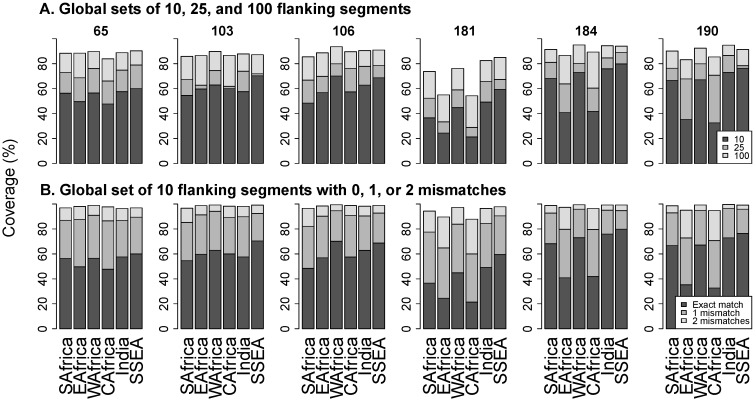
Panel (**A**) shows the proportion of sequences that exactly match the 10 (black bar), 25 (dark grey), and 100 (light grey) most common universal flanking segments overall. Panel (**B**) shows the proportion of sequences that exactly match (black bar), differ by one nucleotide (dark grey), or differ by two nucleotides (light grey) from the 10 most common universal flanking sequences overall. Abbreviations: SAfrica, Southern Africa; EAfrica, East Africa; WAfrica, West Africa; CAfrica, Central Africa; SSEA, South and Southeast Asia.

Figure 3A contains stacked bar plots that show the proportions of flanking segments that exactly match the 10, 25, and 100 most common universal flanking segments pooled from all LMIC regions. The figure shows that the universal set of 10 flanking segments exactly match from a mean of 39.3% sequences at position 181 to 63.2% at position 184. Even the 100 most common flanking segments exactly match from just 71% of sequences at position 181 to 92% at position 184.

Figure 3B contains stacked bar plots that show the proportions of flanking segments that exactly match, differ by one nucleotide, or differ by two nucleotides from a universal set of 10 flanking segments. The proportion of sequences with up to one mismatch with any member of the universal set ranged from a mean of 77% at position 181 to 90% at position 103. The proportion of sequences with up to two mismatches with the set ranged from a mean of 94% at position 181 to 98% at position 184.

**Figure 4:**
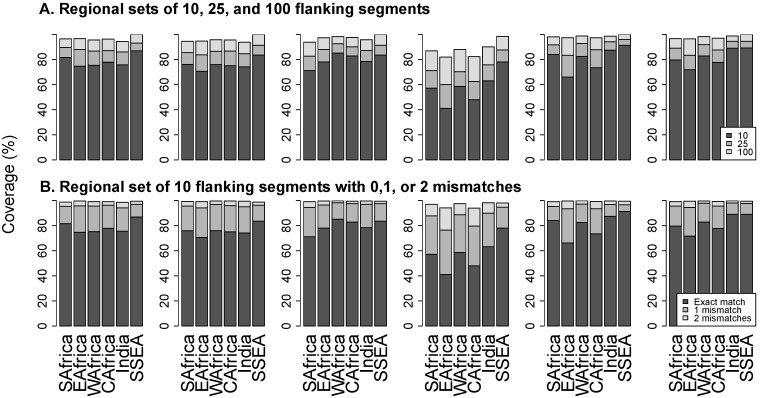
Panel (**A**) shows the proportion of sequences that exactly match the 10 (black bar), 25 (dark grey), and 100 (light grey) most common regional flanking segments. Panel (**B**) shows the proportion of sequences that exactly match (black bar), differ by one nucleotide (dark grey), or differ by two nucleotides (light grey) from the 10 most common regional flanking sequences. Abbreviations: SAfrica, Southern Africa; EAfrica, East Africa; WAfrica, West Africa; CAfrica, Central Africa; SSEA, South and Southeast Asia.

Figure 4A contains stacked bar plots that show the proportions of flanking segments that exactly match the 10, 25, and 100 most common regional flanking segments. The proportion of exact matches for the regional set of 10 flanking segments ranged from a mean of 58% at position 181 to 82% at position 190. The proportion of exact matches for the 100 most common flanking sequences ranged from a mean of 88% at position 181 to 98% at position 184.

Figure 4B contains stacked bar plots that show the proportion of sequences that exactly match, differ by one nucleotide, or differ by two nucleotides from regional sets of 10 flanking segments. The proportion of sequences with up to one mismatch with the set ranged from a mean of 86% at position 181 to 97% at position 106. The proportion of sequences with up to two mismatches with the set ranged from a mean of 97% at position 181 to 99% at position 106.

### 3.4. Online Program

The set of 27,203 RT sequences used for our analysis is available at [24]. An online program that allows users to retrieve: (1) the proportions of codons at a specified position in protease, RT, and integrase according to geographic region and/or subtype; and (2) the proportions of 5' and 3' flanking sequence segments according to segment size, geographic region, and/or subtype is also available at the URL above.

## 4. Discussion

The main challenge in developing hybridization-based point mutation assays for detecting HIV-1 drug resistance mutations is the sequence variability at and surrounding each DRM [11,13]. This genetic variability interfered with the clinical uptake of two previously developed hybridization-based assays: The Affymetrix GeneChip HIV PRT 440 and the Innogenetics INNO-LiPA HIV-1 RT assays [25,26]. However, there has been renewed interest in developing a low cost point-mutation assay for detecting key drug-resistance mutations in LMIC settings [8,9,10,11].

Our analysis characterizes the extent and nature of the sequence variability at and surrounding six candidate POC DRMs by position, subtype, region, and nature of hybridization mismatches. Overall 42 codons at positions 65, 103, 106, 181, 184, and 190 occur in 1% or more sequences of the seven most common subtypes; 13 of these encode the six major DRMs proposed to be most useful for a POC mutation assay. Although the phenotypic effect of these DRMs is likely similar between subtypes, the inter-subtype differences in the surrounding sequences may lead to subtle variations in ARV therapy susceptibilities [27]. Additionally, important differences in codon preference were noted by subtype, including those with clinical implications, and these should be considered in assay development [14,21].

Although the sequence variability surrounding each drug-resistance position may present a more formidable challenge than the variability at the codons of interest, our analysis suggests that most of this variability results from haplotypes that differ from a core set of haplotypes at just one or two positions. Therefore, if the stringency for DRM discrimination can be preserved while allowing for one or two flanking segment mismatches, sensitivity can be increased while maintaining specificity. Our analysis and online program may also identify positions at which degenerate and/or universal bases would be most useful [28,29]. Our analyses also suggest that assays with a flexible design, in that they enabled the use of different probe sets in different regions, would also have increased sensitivity.

## 5. Conclusions

We have described the sequence variability at and surrounding six clinically important HIV-1 DRM positions in a way that identifies several potentially useful strategies for hybridization-based assay development. Additionally, our publicly available online program will allow researchers to perform similar customized analyses to target any HIV-1 DRM position.
